# Case report: A case report on surgical management of laryngeal paraganglioma

**DOI:** 10.1016/j.ijscr.2025.111359

**Published:** 2025-04-23

**Authors:** Melat Teklegiorgis Biru, Bisrat Getachew Yihun, Mezigebu Yaregal Hailu, Addis Asfaw, Hailemariam Kassaye Alebie

**Affiliations:** Department of Otolaryngology-Head and Neck Surgery, St. Paul’s Hospital Millennium Medical College, Addis Ababa, Ethiopia

**Keywords:** Paraganglioma, Laryngeal paragangliomas, Laryngeal tumor, Case report

## Abstract

**Introduction:**

Laryngeal paragangliomas are rare neoplasms originating from neuroendocrine cells within the laryngeal paraganglia.

**Case presentation:**

A 65-year-old woman presented with two years of hoarseness, progressing to dysphagia over the past year, and two months of dyspnea. Laryngoscopy showed a supraglottic mass extending into the right pyriform sinus. Head and neck CT scan revealed a 2 × 3 cm mass, suggestive of laryngeal paraganglioma. Following a discussion of options, she underwent tracheostomy, lateral pharyngotomy and mass excision. Post-operatively, she was asymptomatic. Histopathology confirmed the diagnosis of paraganglioma. The patient was discharged and being monitored regularly. This report follows the SCARE criteria guidelines.

**Clinical discussion:**

Paragangliomas, a slow-growing, benign tumor originating from paraganglia, is a very rare occurrence in the larynx. It typically presents with hoarseness of voice, difficulty of breathing, and difficulty of swallowing. Early diagnosis and surgical intervention are crucial for optimal outcomes.

**Conclusion:**

Laryngeal paraganglioma is a very rare condition. The primary therapeutic option for paraganglioms is complete surgical excision.

## Highlights

• Paragangliomas are rare neuroendocrine tumors arising from extra-adrenal chromaffin cells.
• Laryngeal paragangliomas are a very rare neoplasm.
• Differentiating laryngeal paragangliomas from other laryngeal tumors is challenging.
• Early diagnosis and surgical intervention are crucial for optimal outcomes.
• The primary therapeutic option for paraganglioms is complete surgical excision.

## 1 Introduction

Paragangliomas are rare neuroendocrine tumors arising from extra-adrenal chromaffin cells in sympathetic and parasympathetic ganglia [1]. These tumors are classified as sympathetic or parasympathetic [1,2], most commonly found in the retroperitoneum (55.2 %), head/neck (25.6 %), bladder (5.6 %), and mediastinum (3.2 %) [2,3]. Laryngeal paragangliomas are exceptionally rare, often misdiagnosed as other neoplasms [2], and have a low malignancy rate (1.36 %) [4]. A familial component is present in approximately 35 % of head and neck paragangliomas [4,5].

Clinical presentation varies by location; most are asymptomatic. Laryngeal paragangliomas comprise only 1.41 % of head and neck paragangliomas [3,4], and symptoms are not specific. Hoarseness, for example, can also result from vagal nerve paragangliomas in the parapharyngeal space, particularly those posterior to the styloid process [1]. Differentiating laryngeal paragangliomas from other laryngeal tumors is challenging [2,[4], [5], [6], [7], [8]]. Surgical management, whether conservative or external, presents difficulties in preserving laryngeal and pharyngeal structures and achieving hemostasis [2,[4], [5], [6], [7], [8]].

Imaging, particularly CT and MRI, plays a crucial role in diagnosis [3,4], while histological examination remains the gold standard for definitive diagnosis and differentiation from other cervical neoplasms [4]. Treatment for benign, non-invasive head and neck paragangliomas typically involves surgical resection with wide margins, improving survival rates without adjuvant therapy [[2], [3], [4]].We present here a case of laryngeal paraganglioma who were treated surgically via lateral pharyngeotomy approach.

## 2 Case report

**Fig. 1:**
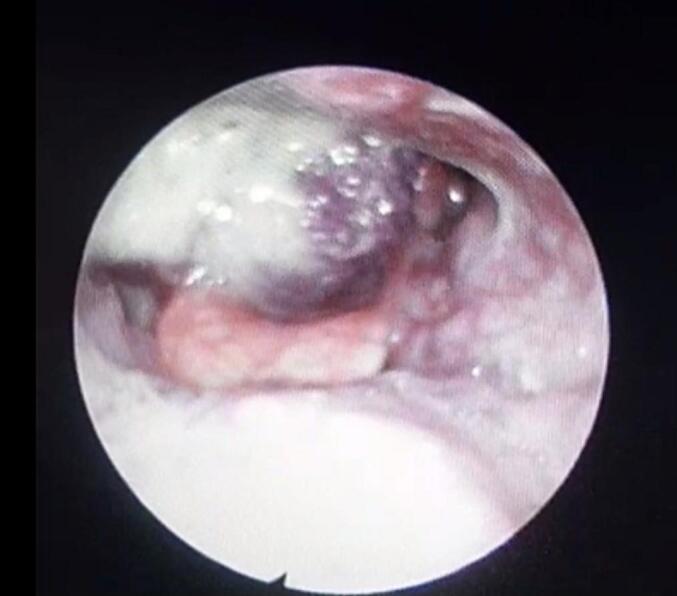
Purplish supraglottic mass extending into the right pyriform sinus.

**Fig. 2:**
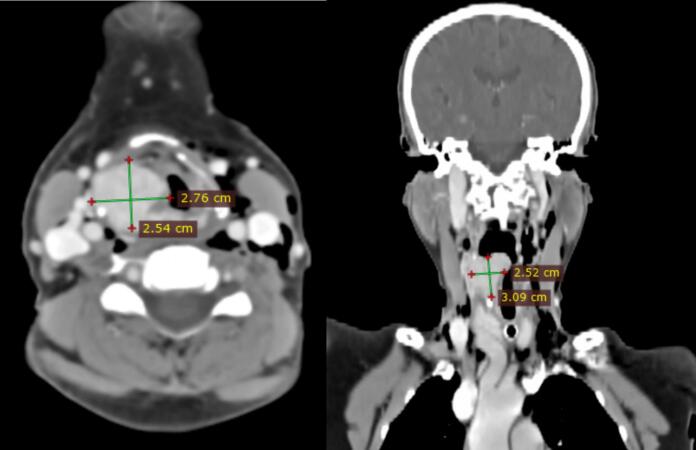
Head and neck CT scan with contrast (Fig. 2) showed a 2 × 3 cm enhancing mass in the right supraglottic region extending to the pyriform sinus.

A 65-year-old female presented with a two-year history of hoarseness of voice and progressive difficulty swallowing over the past year, initially for solids and later liquids. She also reported two months of difficulty breathing. Flexible laryngoscopy (Fig. 1) revealed a purplish supraglottic mass extending into the right pyriform sinus; vocal cord mobility assessment was difficult. Complete blood count (CBC) was normal. Head and neck CT scan with contrast (Fig. 2) showed a 2 × 3 cm enhancing mass in the supraglottic region extending to the pyriform sinus, suggestive of laryngeal paraganglioma.

Following a discussion of treatment options, the patient underwent surgery. An elective tracheostomy was performed for airway management, followed by a right lateral pharyngotomy. A horizontal skin crease incision is made at the level of thyroid cartilage, followed by division of the platysma and elevation of subplatysmal flaps. Vital structures, including the carotid artery, internal jugular vein, and cranial nerves (especially CN X and XII), are identified and preserved. The sternocleidomastoid muscle is retracted laterally to expose the inferior pharyngeal constrictor muscle, and the lateral pharyngeal wall is incised to access the pharyngeal lumen.

**Fig. 3:**
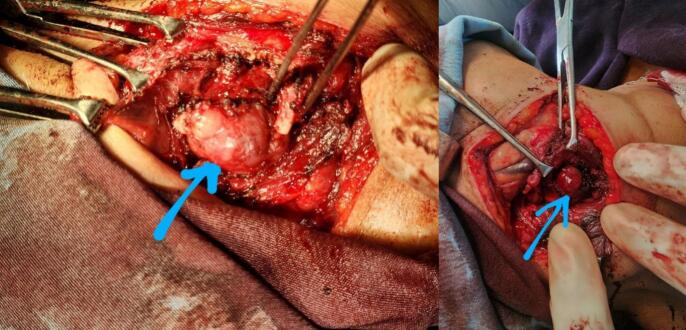
Intraoperative findings included a submucosal, vascular mass originating from the supraglottic region, attached to the thyroid cartilage and hypopharyngeal mucosa.

Intraoperative findings included a submucosal, vascular mass originating from the supraglottic region, attached to the thyroid cartilage and hypopharyngeal mucosa, with a defect in the right thyroid cartilage (Fig. 3). The mass was successfully excised while preserving vital structures. Histopathology result shows well capsulated tumor composed of nests and trabecular of relatively uniform polygonal cells abundant eosinophilic granular cytoplasm, round nuclei and salt and paper chromatin, confirmed the diagnosis of paraganglioma. The well-defined nests of epithelioid cells were arranged in distinct clusters (“Zellballen”) (Fig. 5).

**Fig. 4:**
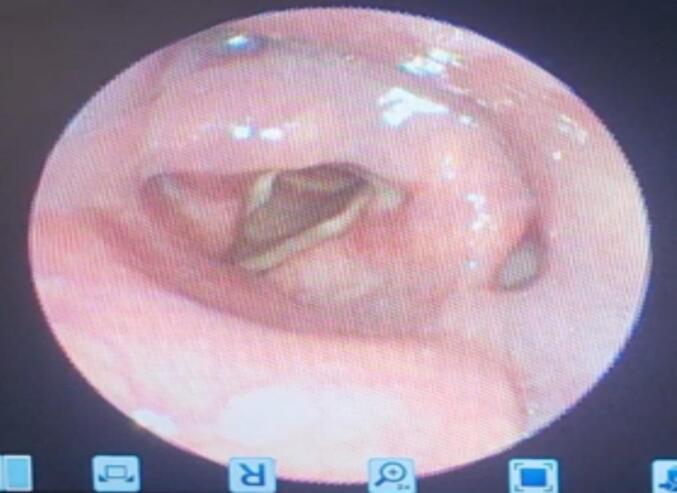
Flexible laryngosopy finding at the first post-operative month, no mass over the larynx.

The postoperative period was uneventful. The patient was discharged with instructions for follow-up and remained asymptomatic at three weeks and two months post-operatively, with resolution of dysphagia and dyspnea. Postoperative flexible laryngosopy finding shows bilateral mobile vocal cord and no visible laryngeal mass (Fig. 4).

**Fig. 5:**
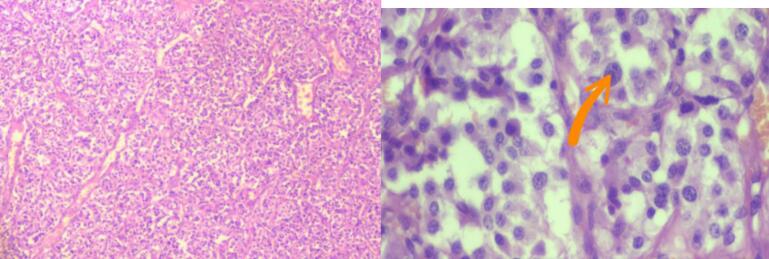
H&E morphology shows Well capsulated tumor composed of nests and trabecular of relatively uniform polygonal cells abundant eosinophilic granular cytoplasm, round nuclei and salt and paper chromatin. The arrow depicts the classic salt and pepper or powdery chromatin of paraganglioma. The well-defined nests of epithelioid cells were arranged in distinct clusters (“Zellballen”).

## 3 Discussion

Paragangliomas are rare neuroendocrine tumors that arise from the extra-adrenal chromaffin cells of the sympathetic and parasympathetic ganglia [1]. These tumors are classified into two groups: head and neck tumors, which arise from the parasympathetic nervous system and do not produce catecholamines, and sympathetic paragangliomas, which occur along the axis of the body from the skull base to the pelvic floor and secrete catecholamines [1].

The larynx contains two pairs of paraganglia: superior and inferior. The superior laryngeal paraganglia are located on the edge of the thyroid cartilage, while the inferior laryngeal paraganglia can be found anywhere from the inferior cornu of the thyroid cartilage to the cricoid cartilage [2,3]. Laryngeal paragangliomas are the least common type of head and neck paragangliomas, representing only 1.41 % of all cases. Patients with laryngeal paragangliomas typically present with symptoms such as dyspnea, dysphagia, hoarseness, chronic cough, and stridor. Most of these tumors are non-functional and do not show symptoms associated with elevated catecholamine levels [4].

In our case, the patient presented with significant voice changes and respiratory distress, emphasizing the importance of including paragangliomas in the differential diagnosis of laryngeal tumors. Histological and immunohistochemical examinations are the gold standard for diagnosing laryngeal paragangliomas, as they help differentiate them from other neoplasms, such as carcinoid tumors or schwannomas. Although these tests cannot definitively determine whether the tumor is benign or malignant, they provide diagnostic clues by identifying the “Zellballen” pattern and measuring Ki67 [5,6].

Management of laryngeal paragangliomas primarily involves surgical excision, with the extent of resection depending on the tumor's size and local involvement [4]. Open surgical procedures have shown higher disease-control rates. In earlier reports, some patients underwent total laryngectomy [7].

Transoral endoscopic approach is limited by reduced access and difficulty in controlling bleeding, which can obscure the narrow surgical field. It has higher recurrence rates and has largely been replaced by the external cervical approach. Endoscopic techniques are restricted to the endolarynx and are not suitable for paragangliomas extending beyond it [10]. Other described methods include microlaryngoscopy with laser excision and transoral robotic surgery; however, neither has surpassed the external approach. The external cervical approach remains preferred due to its superior access, visualization, and effective hemorrhage control [9].

Adjuvant therapies, including radiation, may be considered for cases with significant residual disease, although their role is less well-defined [5,7]. In this case, complete surgical resection led to a favorable outcome, with no recurrence observed at the six-month follow-up.

Prolonged surveillance is crucial. The British Skull Base Society recommends an MRI scan every six months initially, with annual re-examinations afterward. There is no universal consensus on the duration of surveillance. The British Skull Base Society suggests frequent imaging during the first three years, followed by longer intervals, while the European Society of Endocrinology advises a minimum of 10 years of monitoring, with lifelong observation for patients with genetic predispositions or risk factors [8].

## 4 Conclusion

In conclusion while laryngeal paragangliomas are rare, they should be considered in the differential diagnosis of laryngeal tumors, especially in patients presenting with hoarseness and airway compromise. Early-career clinicians should recognize that although rare, laryngeal paragangliomas must be considered when diagnosing vascular masses in the larynx. Comprehensive histopathologic examination and immunohistochemical profiling are essential for diagnosis, and a multidisciplinary approach ensures optimal management. Future research should focus on better understanding the pathogenesis and optimal treatment modalities for this intriguing sub type of paraganglioma.

## Previous publications

1. Primary Tuberculosis Otomastoiditis in a 25-Year-Old Male: An Ethiopian Case Report DOI:[10.61148/2836-2845/ISCR/075](doi:10.61148/2836-2845/ISCR/075)
2. A Case Report on Adult-Onset Cystic Hygroma and Literature Review DOI:[https://doi.org/10.1016/j.ijscr.2024.110595](doi:10.1016/j.ijscr.2024.11055)
3. A Case Report on Surgical Management of Glomus Tympanicum and Literature Review DOI: [https://doi.org/10.1016/j.ijscr.2025.11084]

## Informed consent

Informed consent was obtained from the patient for their anonymized information to be published in this article.

## Ethical approval

Our institution does not require ethical approval for reporting individual cases or case series.

## Guarantor

Melat Teklegiorgis Biru.

## Funding

No financial support for the research, authorship, and/or publication of this article.

## Declaration of competing interest

The authors have no financial conflicts of interest.

## Data Availability

All data are included within the article.
